# Supplementary figures and images for: Genomic epidemiology of Campylobacter jejuni associated with asymptomatic pediatric infection in the Peruvian Amazon
Source: PLoS Negl Trop Dis. 2020 Aug 10;14(8):e0008533. doi: 10.1371/journal.pntd.0008533 (PMC7440661; doi:10.1371/journal.pntd.0008533)

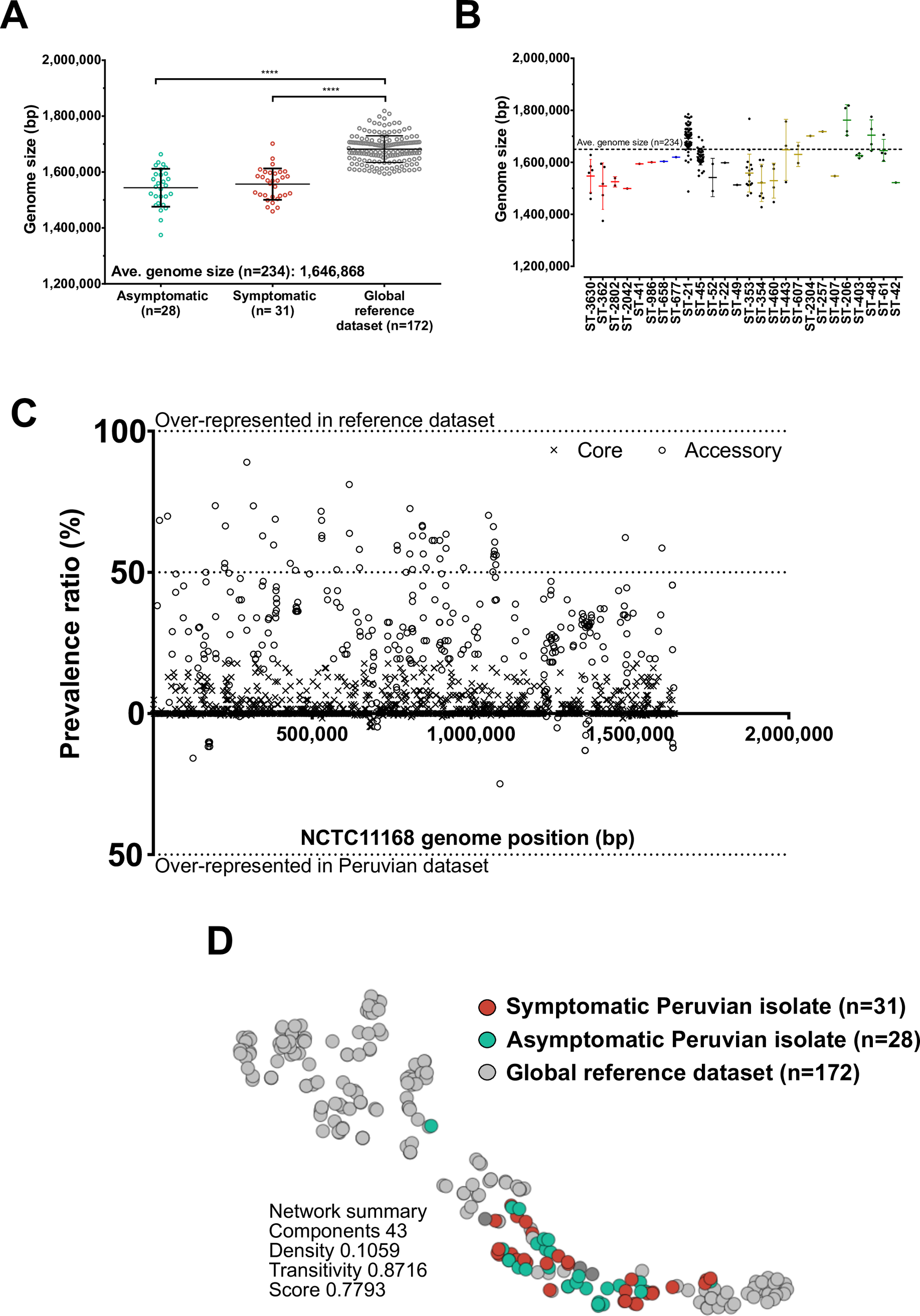

Supplement: S1 Fig — Genome size comparisons between (A) Asymptomatic (green) and symptomatic (red) Peruvian isolate genomes with the reference dataset (grey); and (B) all sequence types (ST) represented by 3 or more genomes in the dataset. Dotted line indicates the average genome size for all isolates in the dataset (1,646,868 bp). (C) Relative presence of all NCTC 11168 genes (n = 1,623) in the Peruvian and reference datasets. Genes core and accessory in the reference dataset are indicated by (x) and (o), respectively. Genes present more often in one dataset compared to the other appear further from the mid-line. (D) Pairwise core and accessory genome distances were compared using PopPunk for the Peruvian genomes and full dataset (version 1.1.4) [70]. Clustering visualized using the t-distributed stochastic neighbor embedding (t-SNE) projection of accessory distances in microreact. (TIF) [file pntd.0008533.s010.tif]

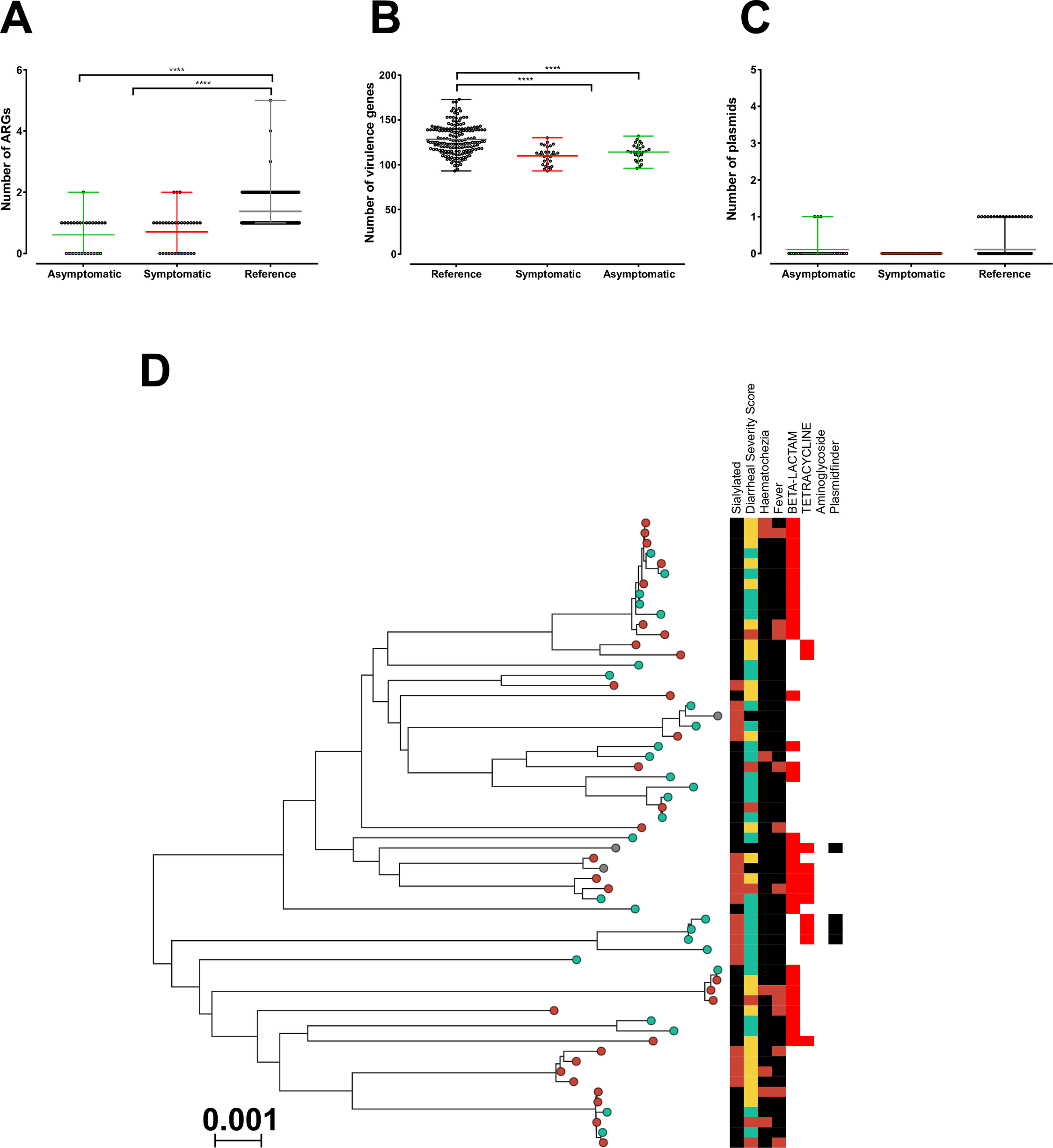

Supplement: S2 Fig — Number of (A) antimicrobial resistance genes (ARGs), (B) virulence genes and (C) predicted plasmids per isolate estimated using ABRICATE (version 0.9.8; [69]). (D) Maximum-likelihood phylogeny of the Peruvian isolates only. The tree is annotated with disease severity scores, the onset of specific symptoms (hematochezia and fever), presence of AMR genes (beta-lactams, tetracyclines or aminoglycosides), identified plasmids and sialylation prediction. (TIF) [file pntd.0008533.s011.tif]

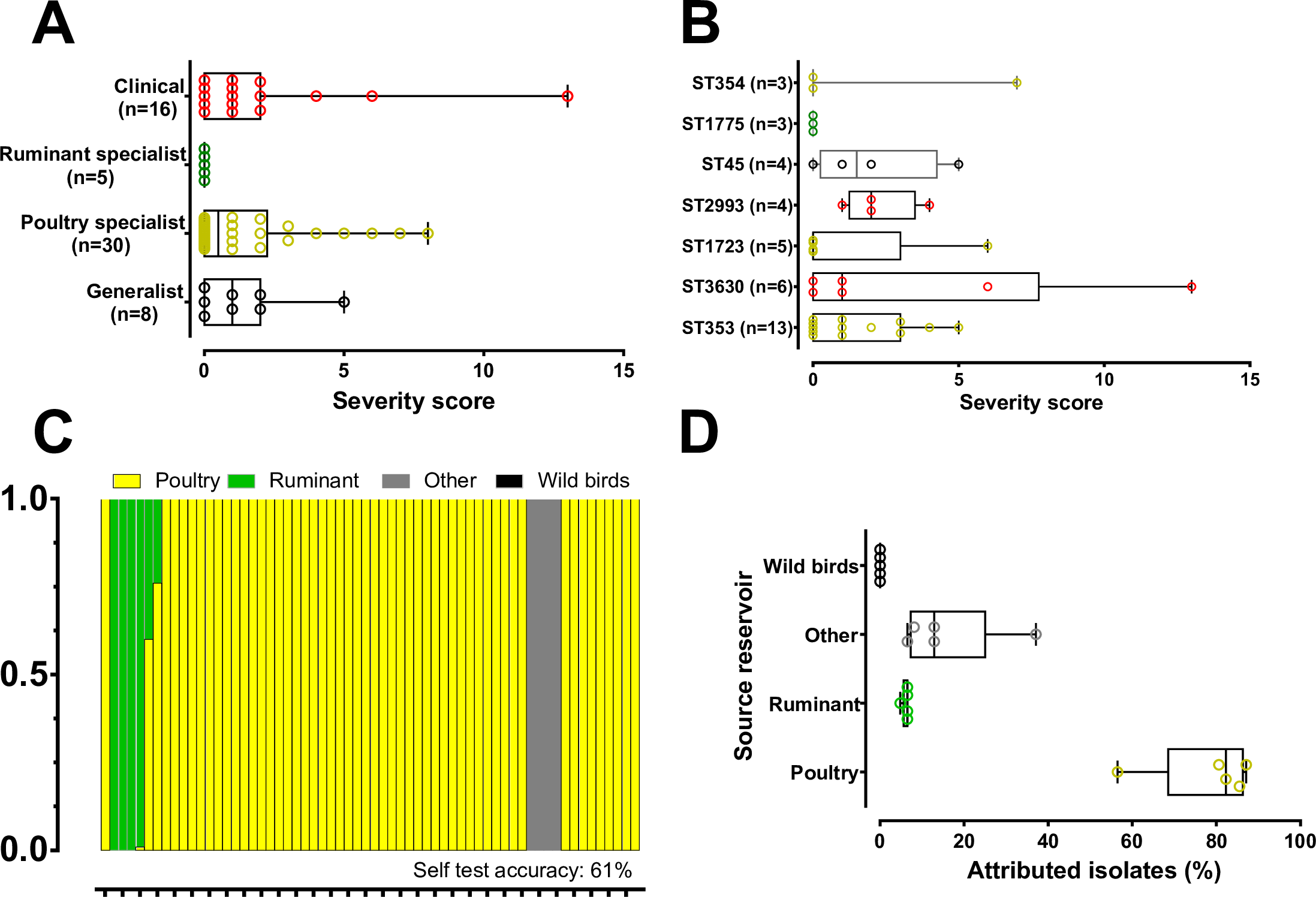

Supplement: S3 Fig — Average disease severity score by (A) isolate host ecology and (B) sequence type (represented by 3 or more isolates). (C) Representative source attribution of Peruvian pediatric isolates using the Bayesian clustering algorithm STRUCTURE (version v2.3.4, [71]). Each isolate is represented by a vertical bar colored by the estimated probability that it originated from putative source reservoirs (yellow: chicken; green: ruminant; black: wild bird and grey: other). (D) Summary box plots of predicted attribution of 62 Peruvian pediatric isolates following 5 independent estimations. (TIF) [file pntd.0008533.s012.tif]
